# Mini-Percutaneous Nephrolithotomy With an Endoscopic Surgical Monitoring System for the Management of Renal Stones: A Retrospective Evaluation

**DOI:** 10.3389/fsurg.2022.773270

**Published:** 2022-07-11

**Authors:** Huiming Gui, Hanzhang Wang, Dharam Kaushik, Ronald Rodriguez, Zhiping Wang

**Affiliations:** ^1^ Department of Urology, Institute of Urology, Gansu Nephro-Urological Clinical Center, Key Laboratory of Urological Diseases in Gansu Province, Lanzhou University Second Hospital, Lanzhou, China; ^2^ Department of Urology, University of Texas Health Science Center at San Antonio, San Antonio, TX United States

**Keywords:** standard percutaneous nephrolithotomy, mini-percutaneous nephrolithotomy, endoscopic surgical monitoring system, renal calculus, retrospective study

## Abstract

**Purpose:**

To compare the outcomes and postoperative quality of life of patients with renal calculi who underwent standard percutaneous nephrolithotomy (sPNL), mini-invasive percutaneous nephrolithotomy (mPNL) or mPNL with an endoscopic surgical monitoring system (ESMS) using a retrospective clinical trial.

**Methods:**

Eighty-six adult patients with renal stones who were treated with sPNL were retrospectively compared to ninety-two patients who were treated with mPNL between July 2014 and December 2017. Next, further studies were retrospectively conducted using a matched paired method. The ninety-two patients treated with mPNL were divided into two groups based on whether the endoscopic surgical monitoring system (ESMS) was used (ESMS-mPNL vs. non-ESMS-mPNL). The ESMS used strain gauge transducers to measure the inflow and outflow of irrigation solution. Bleeding and fluid absorption during endoscopic surgery could be accurately calculated by computer program in ESMS.

**Results:**

The fluoroscopy time, complication rate, stone-free status and clinically insignificant residual fragment (CIRF) rate were not significantly different between the two groups (sPNL vs. mPNL). The mPNL group had a significantly longer operation time than the sPNL group, and the mPNL group exhibited a markedly reduced 12-h postoperative visual analogue pain scale (VAS) score, mean hospitalization time, and return to work time, had slightly reduced haemoglobin loss, and underwent more tubeless operations. Moreover, among the 92 patients who underwent mPNL, the operation time (*P *= 0.090), complication rate (*P *= 0.996), stone-free status (*P *= 0.731), CIRF rates (*P *= 0.125) and number of tubeless operations (*P *= 0.760) were not significantly different between the two subgroups (non-ESMS-mPNL vs. ESMS-mPNL); however, the patients in the ESMS-mPNL group had significantly longer irrigation times than those in the non-ESMS-mPNL subgroup, along with marked reductions in irrigation fluid, blood loss, haemoglobin loss, 12 h postoperative VAS score, mean hospitalization time, and return to work time.

**Conclusions:**

mPNL is less painful than sPNL in patients undergoing treatment for 20–40 mm renal stones. Similar stone-free rates were achieved by the two procedures, but mPNL was superior to sPNL in terms of blood loss, discomfort, hospitalization time and return to work time. We think that ESMS-mPNL is less painful for patients and more efficacious than non-ESMS-mPNL, and ESMS-mPNL achieves a stone-free rate that is similar to non-ESMS-mPNL in patients receiving treatment for 20–40 mm kidney stones.

## Introduction

Percutaneous nephrolithotomy (PNL) should be the most commonly used first-line treatment for patients with large or complex renal stones (1); however, PNL can cause serious complications and morbidities, including bleeding, organ injury, pain, infection, vascular embolism and accidental death (2, 3). Therefore, there is a need for alternative treatments that minimize the risks associated with PNL (4). Mini-PNL (mPNL) was originally used for paediatric patients, and later, it was widely applied to the general population because it can reduce complications and morbidities (5).

In the last 20 years (with the development of minimally invasive nephroscopy, nephrostomy sheath and computer imaging technology), sPNL has been partially replaced by mPNL (6). However, whether mPNL is more effective and safer than sPNL is still inconclusive, and the debate is ongoing. Ruhayel et al. (1) confirmed that mPNL can achieve a considerable stone-free rate (SFR), but the operation time is longer. However, mPNL has the obvious advantages of reduced bleeding and a shorter hospital stay. Jiao et al. (7) demonstrated that the overall evidence was not sufficient to prove a significant difference between mPNL and sPNL in terms of complications and morbidities.

Some studies have reported that operation with a continuous open flow system using X-ray or endoscopic guidance can also be used to prevent electrolytic imbalance. When the difference between the inflow and outflow fluid exceeds 500 ml, further procedures should be terminated, a nephrostomy tube must be used, and the electrolyte levels need to be measured (8, 9). Endoscopic monitoring is also helpful to evaluate changes in irrigating fluid absorption, hemodynamics and electrolyte levels (10). We measured irrigation fluid absorption and bleeding with a new strategy called the endoscopic surgical monitoring system (ESMS). The use of the ESMS to guide percutaneous renal access during mPNL has never been reported; hence, we performed a retrospective study to assess the safety and efficacy of ESMS-guided renal access in PNL.

It is still uncertain whether mPNL with the use of an endoscopic surgical monitoring system is superior to mPNL or sPNL; hence, we performed a retrospective study comparing the outcomes of the three major surgical techniques currently used in patients with kidney stones.

## Methods

### Patients and Grouping

This retrospective study was approved by the Ethical Committee of the Lanzhou University Second Hospital (No. 2016A-059; Date: July 20th, 2016), and patients signed an informed consent form before the operation. Between June 2014 and November 2017, 354 patients underwent surgery for renal calculi.

Demographic data, patient history, symptoms and signs, image analyses, ultrasound data, and surgical procedures were collected through chart review. In addition, postoperative clinical data were collected through chart review and outpatient records.

**Figure 1:**
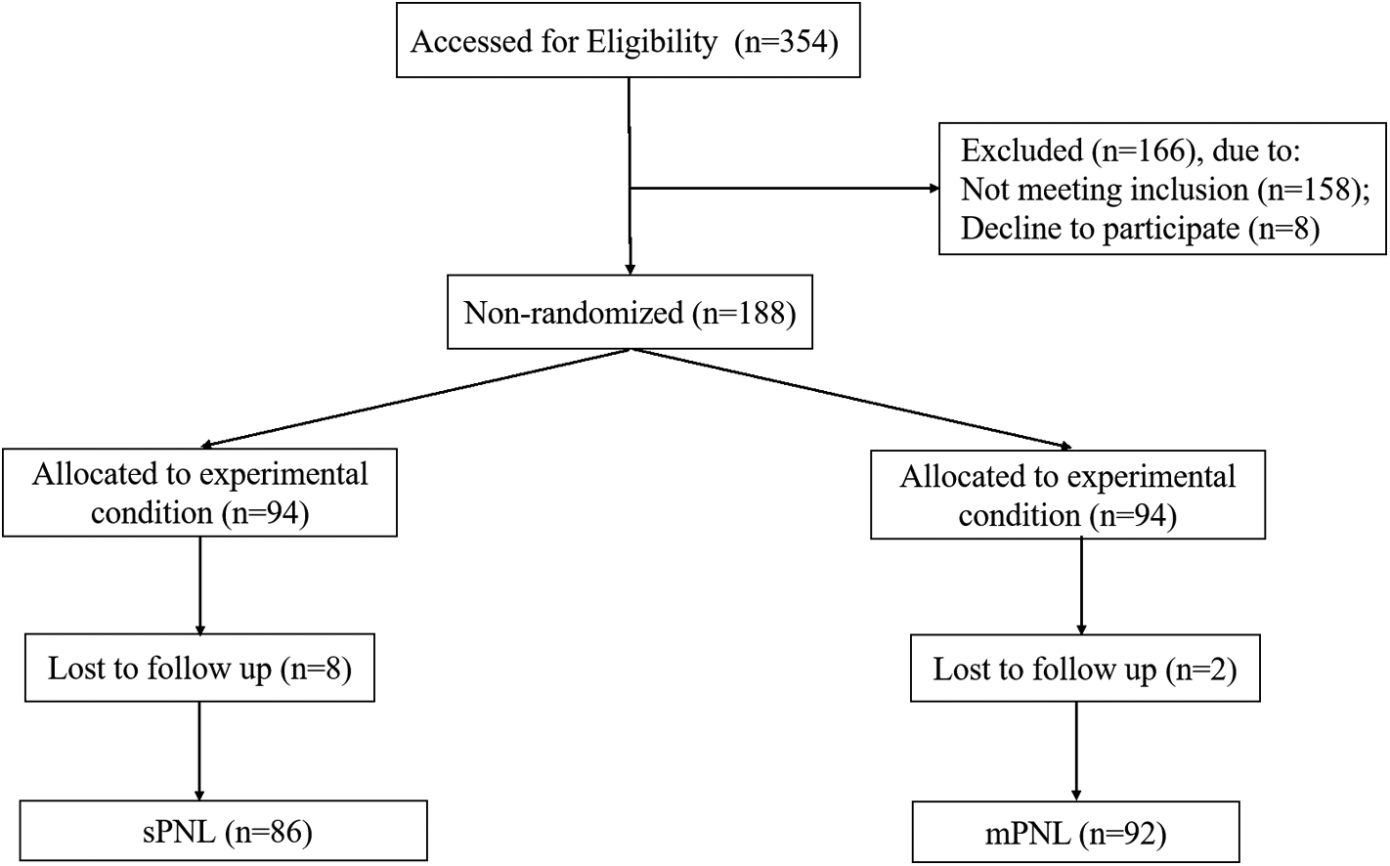
Specific procedures for case screening and patient grouping.

The study design and workflow are summarized in Figure 1. We selected the appropriate patient for each procedure according to the patient's preference. A total of 178 patients were divided into two groups based on surgical procedure. Group 1 included 86 patients who underwent sPNL, while Group II included 92 patients who underwent mPNL. The ninety-two patients with renal stones who underwent mPNL were further divided into two subgroups based on whether the endoscopic surgical monitoring system was used during mPNL (ESMS-mPNL): Group III (ESMS-mPNL, 46) and Group IV (non-ESMS-mPNL, 46).

The exclusion criteria were age < 18 years or > 65 years and congenital renal abnormalities, solitary kidney or hydronephrosis, impaired renal function or coagulation disorder. In the preoperative period, all the patients were evaluated by urinalysis, urine culture, coagulation tests and radiologic studies, including ultrasonography, radiography of the kidney, ureter, and bladder (KUB) and computerized tomography (CT), and the haemoglobin (Hgb), serum urea and creatinine levels of the patients were measured. The stone sizes were determined by measuring the longest axis of the stones on radiology.

### Surgical Techniques

All the patients were assessed by noncontrast CT (NCCT) before the operation. The size of each stone was determined by measuring the maximum size of each stone. For multiple stones, the sum of the maximum size of each stone was calculated. All patients with preoperative urinary infections were treated with antibiotics based on the bacterial culture and sensitivity tests. When the urine cultures became sterile, the patients were scheduled for PNL. For antibiotic prophylaxis, second-generation cephalosporins were administered before surgery, and the antibiotics were continued until the nephrostomy catheter was removed. Percutaneous renal puncture under fluoroscopic guidance was performed with patients in the prone position.

For patients undergoing sPNL, the nephrostomy tract was dilated up to 22–30 F using an Alken metal expander (Karl Storz, Tuttlingen, Germany), followed by the placement of the Amplatz sheath. A 24-F nephroscope (R. Wolf, Knightlingen, Germany) was applied. Finally, the stones were fragmented by pneumatic lithotriptor (Swiss lithotriptor EMS, Switzerland), and the fragments were removed using a grasper. When the operation was almost complete, a ureteral catheter was sometimes placed based on the intraoperative findings and the decision of the surgeon.

For the mPNL procedure, the bundle was expanded to 18–20 F using a single-step expander under spinal anaesthesia. A 12-F rigid nephroscope was used. A pneumatic ballistic lithotripsy was used to break the stones. During the removal of the nephroscope, the stone fragments were removed through the ureteral catheter. When the operation was nearly complete, the ureteral stent was placed and the sheath was directly led out by visual inspection. The nephrostomy tube (no tube) was not inserted if there were no complications (e.g., bleeding, perforation of the renal calyceal system) or presence of obvious residual stones, and the patient was not scheduled for a second examination. The kidneys were continuously rinsed with NaCl solution (0.9%), and the absorbed fluid volume was worked out according to previously described criteria (11). Briefly, the volumes of total irrigation fluid used and total drainage fluid, including the fluid found on the floor and in the curtain, were measured, and the difference between them was considered as the volume of absorbed liquid.

**Figure 2:**
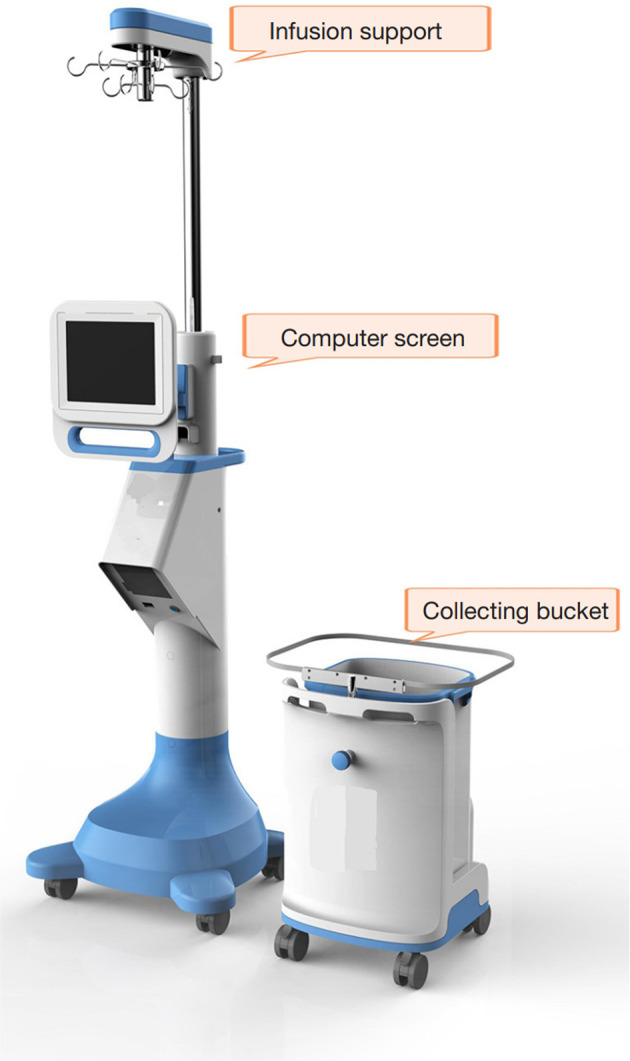
The prototype of endoscopic surgical monitoring system.

**Figure 3:**
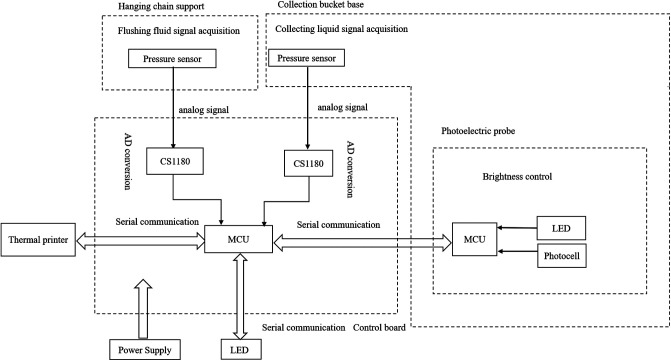
The working principle of ESMS is used in PNL. CS1180: high precision conversion chip; LED: light emitting diode; MCU: micro control unit.

**Figure 4:**
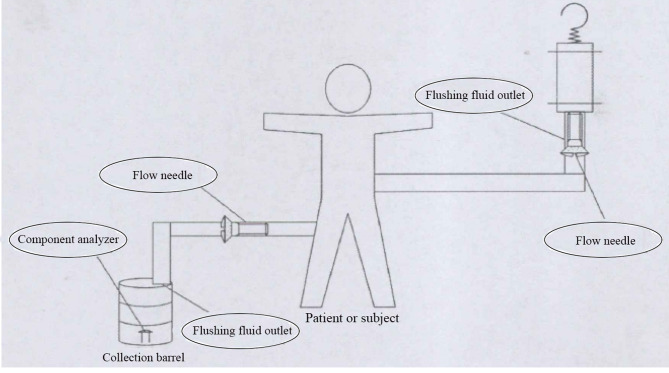
Measurement of fluid absorption in ESMS-mPNL patients.

The endoscopic surgical monitoring system (ESMS) was patented and approved by the Chinese Food and Drug Administration, and this system is starting to be produced (approved no: 20162210011) (Figure 2). The working principle of the ESMS is illustrated in Figure 3. The ESMS was confirmed to be accurate and valid during urological endoscopic surgery (10). For the mPNL procedure, an 18 G percutaneous needle was used to enter the renal collection system under ultrasound guidance. The renal collection system was dilated to 20 F with a fascia expander, and a stripping sheath was placed. The fragmentation and removal of stones were performed by a rigid nephroscope passing through the sheath. Continuous irrigation of the kidney was performed with normal saline at room temperature (22°C), and an automatic pumping irrigation system was used to maintain a fixed pressure. All the patients were administered intravenous fluids with lactated Ringer's solution. The hemoglobin, haematocrit, electrolyte, urea and creatinine levels were measured 10 min before the operation for postoperative comparison. The irrigation time, volume of irrigation fluid used, blood loss and irrigation fluid absorption were monitored by the ESMS. The process of liquid absorption measurement for patients who underwent surgery with the ESMS is shown in Figure 4.

### Follow-up

The visual analogue scale (VAS) was used to assess the patient's pain after the operation (11). In our clinic, we routinely perform VAS measurements in the postoperative period. The VAS was used to classify pain severity of ten 1-cm horizontal segments, with 0 cm indicating no pain and 10 cm indicating the worst pain. The VAS scores were assessed at 12 h postoperatively (12). The fluoroscopy time (FT), operation time (OT), JJ stent insertion rate, hospitalization time (HT) and return to work time (RWT) were also noted. Complications were classified based on the Clavien classification system (13).

On the first postoperative day, the general condition and pain status of the patients were evaluated, and the KUB was evaluated to verify JJ stent insertion and to verify that the patient had a stone-free status. During the first postoperative month, low-dose computed tomography was performed. A stone-free status was defined as no residual debris on CT evaluation during the first month after the operation. Residual stones ≤4 mm in size were defined as clinically insignificant residual fragments (CIRFs). After obtaining approval from the local ethics committee, we retrospectively assessed the patient files and documents in our clinics. An informed consent form including the ethical information and the detailed surgical procedures was given to all the patients before the surgery.

### Statistical Analysis

All statistical analyses in this retrospective study were conducted using SPSS 26.0. The complication rate, stone-free rate, CIRF rate, and number of tubeless procedures, as well as other perioperative variables, were compared between the two groups (sPNL vs. mPNL) using Student's t test. A logistic regression analysis was performed to compare the fluoroscopy time, operative time, haemoglobin loss, mean hospitalization time, and return to work time between the groups. The analysed factors also included the mean age, sex, stone size, BMI, history of previous open renal surgery (ORS), stone characteristics (number, size, localization), hydronephrosis grade, and whether sPNL or mPNL was performed. Baseline characteristics and perioperative parameters in both subgroups (ESMS-mPNL vs. non-ESMS-mPNL) were compared by means of paired, Tukey's, and independent t tests. *P* values <0.05 were considered statistically significant.

## Results

### Patient Recruitment and Clinical Features

In the present study, a total of 178 patients (86 underwent sPNL and 92 underwent mPNL) were enrolled. The mean age of the patients was 40.42 ± 11.68 and 41.93 ± 11.9 years in the sPNL group and the mPNL group, respectively. Table 1 summarizes the preoperative data analysis of all the enrolled patients. Clinical parameters did not differ significantly between the groups (mPNL vs sPNL), including mean age, sex, BMI, stone size, number of stones, side of surgery, stone localization, and preoperative haemoglobin. However, the previous ORS of the patients were significantly higher in the mPNL group.

**Table 1:** Preoperative data of all patients.

Demographic data	sPNL (*n* = 86)	mPNL (*n* = 92)	*P* value
The mean age, mean ± SD	40.42 ± 11.68	41.93 ± 11.9	0.395
Gender (male/female)	56/30	62/30	0.750
The mean stone size (mm), mean ± SD	29.0 ± 5.32	28.74 ± 4.93	0.735
BMI (kg/m^2^)	24.7 ± 3.54	24.4 ± 4.02	0.599
Previous ORS	9/86	22/92	0.020
Number of stones			
1	71	72	0.473
≥2	15	20	
Side of surgery			
Left	45	46	0.758
Right	41	46	
Location of stone (%)			
Upper pole	35	32	0.991
Middle pole	25	36	
Lower pole	26	24	
Hydronephrosis grade, *n* (%)			
G0	15	17	0.998
Mild (G1 or G2)	30	28	
Moderate (G3)	20	22	
Severe (G4)	21	25	

*BMI*, *body mass index; G0*, *grade 0; G1*, *grade 1; G2*, *grade 2; G3*, *grade 3; G4*, *grade 4; mPNL*, *mini-PNL; ORS, open renal surgery; PNL*, *percutaneous nephrolithotomy; SD*, *standard deviation; sPNL*, *standard PNL.*

We retrospectively compared 92 mPNL patients, including 46 in the ESMS-mPNL group and 46 patients in the non-ESMS-mPNL subgroup. The operative data are presented in Table 2. No difference was detected between the groups (ESMS-mPNL vs non-ESMS-mPNL) regarding mean age, sex, BMI, previous ORS, preoperative haemoglobin, number of stones, side of surgery, stone location, or hydronephrosis grade.

**Table 2:** Preoperative data of 92 mPNL patients.

Demographic Data	Non-ESMS-mPNL (*n* = 46)	ESMS-mPNL (*n* = 46)	*P* value
The mean age ± SD, mean ± SD	41.36 ± 12.32	42.08 ± 12.66	0.781
Gender (male/female)	34/12	28/18	0.182
The mean stone size (mm), mean ± SD	29.22 ± 5.36	28.26 ± 4.38	0.349
BMI (kg/m^2^)	25.3 ± 3.67	24.5 ± 4.05	0.319
Previous ORS	10/46	12/46	0.631
Preoperative hemoglobin (gm/dL)	12.39 ± 1.21	12.94 ± 1.62	0.070
Number of stones			
1	35	37	0.613
≥2	11	9	
Side of surgery			
Left	22	24	0.677
Right	24	22	
Location of stone (%)			
Upper pole	16	16	0.287
Middle pole	15	21	
Lower pole	15	9	
Hydronephrosis grade, *n* (%)			
G0	9	8	0.896
Mild (G1 or G2)	14	14	
Moderate (G3)	12	10	
Severe (G4)	11	12	

*BMI*, *body mass index; ESMS*, *endoscopic surgical monitoring system; G0*, *grade 0; G1*, *grade 1; G2*, *grade 2; G3*, *grade 3; G4*, *grade 4; mPNL*, *mini-PNL; ORS*, *open renal surgery; PNL*, *percutaneous nephrolithotomy; SD*, *standard deviation; VAS, visual analogue scale*.

### sPNL vs. mPNL for Renal Stones

The complications and postoperative outcomes of the patients in the sPNL group vs. the mPNL group are presented in Table 3. A shorter fluoroscopy time was reported in the mPNL group (118 ± 13.0 vs. 122 ± 14.1 s), which was not statistically significant (*P* = 0.051). The CIRF rate and SFR at 1 month were both similar between the two groups. Clavien grade 1 complications were comparable between the groups, 3 patients in the sPNL group vs 2 patients in the mPNL group. In both the sPNL and mPNL groups, 1 patient had a Clavien grade 2 complication and received transfusions. In terms of postoperative complications, no difference was observed between the groups (*P* = 0.182).

**Table 3:** Operative, postoperative and outcomes of sPNL and mPNL.

Data	sPNL (*n* = 86)	mPNL (*n* = 92)	*P* value
Fluoroscopy time (sec.), mean ± SD	122 ± 14.1	118 ± 13.0	0.051
Operation time (min.), mean ± SD	57.3 ± 7.5	67.4 ± 8.1	<0.001
Hemoglobin loss (mg/dl)	1.46 ± 0.93	1.14 ± 0.74	0.012
VAS score postop 12 h, mean ± SD	2.05 ± 0.48	1.82 ± 0.54	0.003
Complications rate			
Clavien 1	3 (5.2)	2 (4.1)	0.182
Clavien 2	1 (1.9)	1 (1.6)	
Clavien 3	–	–	
Clavien 4	–	–	
Mean hospitalization time (hour), mean ± SD	53.47 ± 13.21	49.27 ± 11.34	0.024
Stone-free rate (1. month)	75 (89.1)	80 (90.3)	0.961
CIRF rate (%)	2 (2.3)	3 (3.3)	0.71
Return to work time (day), mean ± SD	12.16 ± 2.41	10.98 ± 2.48	0.002
Tubeless procedure (%)	4 (4.7)	34 (37.0)	<0.001

*CIRF*, *clinically insignificant residual fragment; mPNL*, *mini-PNL; PNL*, *percutaneous nephrolithotomy; SD*, *standard deviation; VAS*, *visual analogue scale.*

A longer operative time was reported with the mPNL group (67.4 ± 8.1 vs. 57.3 ± 7.5 min, respectively; *P* < 0.001). The amount of haemoglobin loss was significantly reduced in the mPNL group (1.46 ± 0.93 vs. 1.14 ± 0.74, respectively; *P* = 0.012), and the 12 h postoperative VAS score was lower in the mPNL group (2.05 ± 0.48 vs. 1.82 ± 0.54, respectively; *P* = 0.003). We found that the patients in the mPNL group had significant reductions in the return-to-work time (*P* = 0.002) and the hospitalization time (*P* = 0.024). Thirty-four (37.0%) patients in the mPNL group and 4 (4.7%) patients in the sPNL group were treated by tubeless surgery (*P *< 0.001).

### ESMS-mPNL vs. non-ESMS-mPNL for Renal Stones

The complications and postoperative outcomes of ESMS-mPNL vs. non-ESMS-mPNL are presented in Table 4. A longer operation time was reported with ESMS-mPNL (66.1 ± 6.2 vs. 68.2 ± 5.6 min), which was not statistically significant (*P* = 0.090). The CIRF rate and stone-free rate at 1 month were both similar between the two subgroups (*P* = 0.125). Eighteen (39.1%) patients in the non-ESMS-mPNL group and 16 (34.8%) in the ESMS-mPNL group underwent tubeless surgeries (*P *= 0.670). Two patients had Clavien grade 1 complications, and both patients received transfusions. In terms of the postoperative complications, no difference was found between the groups (*P* = 0.996).

**Table 4:** Comparison of operative data and complications for Non-ESMS-mPNL vs ESMS-mPNL groups.

Data	Non-ESMS-mPNL (*n* = 46)	ESMS-mPNL (*n* = 46)	*P* value
Operation time (min.), mean ± SD	66.1 ± 6.2	68.2 ± 5.6	0.090
Irrigation time (min)	42.2 ± 14.1	52.0 ± 18.3	0.005
Volume of irrigation fluid (ml)	1651.9 ± 631.4	1245.6 ± 548.2	0.001
Volume of fluid absorbed (ml)	712 ± 95	502 ± 102	<0.001
Blood loss (ml)	142.1 ± 93.54	82.2 ± 41.2	<0.001
Hemoglobin loss (mg/dl)	1.21 ± 0.78	1.02 ± 0.63	0.044
VAS score postop 12 h	1.95 ± 0.56	1.66 ± 0.42	0.005
Complications rate			
Clavien 1	2 (4.8)	2 (3.2)	0.996
Clavien 2	–	–	
Clavien 3	–	–	
Clavien 4	–	–	
Mean hospitalization time (hour), mean ± SD	53.82 ± 13.48	47.31 ± 12.04	0.017
Stone-free rate (1. month)	41 (89.1)	42 (90.3)	0.731
CIRF rate (%)	2 (4.3)	1 (2.2)	0.125
Return to work time (day), mean ± SD	12.06 ± 3.21	9.87 ± 2.76	0.001
Tubeless procedure (%)	18 (39.1)	16 (34.8)	0.670

*CIRF*, *clinically insignificant residual fragment; ESMS, endoscopic surgical monitoring system; mPNL*, *mini-PNL; PNL*, *percutaneous nephrolithotomy; SD, standard deviation; VAS = visual analogue scale.*

The comparison of the laboratory values in the patients in the ESMS-mPNL and non-ESMS-mPNL subgroups showed a significant decrease in irrigation fluid absorption (*P* = 0.001), blood loss (*P* < 0.001), and haemoglobin loss (*P* = 0.044) (Table 4). A longer irrigation time (52.0 ± 18.3 vs. 42.2 ± 14.1 min) and a smaller volume of absorbed fluid (502 ± 102 vs. 712 ± 95 ml) were observed in the patients in the ESMS-mPNL group compared with those in the non-ESMS-mPNL group (*P* = 0.005 and *P* < 0.001, respectively).

The mean hospitalization time in the non-ESMS-mPNL subgroup was 53.82 ± 13.48, compared to 47.31 ± 12.04 in the ESMS-mPNL subgroup; these values were significantly different (*P* = 0.017). The mean return to work time was 12.06 ± 3.21 in the non-ESMS-mPNL subgroup and 9.87 ± 2.76 in the ESMS-mPNL subgroup, which was a significant difference (*P* = 0.001).

## Discussion

Numerous studies debate the merits of minimally invasive PNL (14, 15), and there are considerable debates regarding the merits of mPNL and sPNL (16–18). Irrigating fluid absorption, bleeding and haemodynamic abnormalities are common. PNL-related complications are common, and patient recovery from anaesthesia is challenging, especially in high-risk groups (19, 20). There are few studies on PNL-related blood loss, haemodynamic changes and electrolyte levels. Similarly, there are only a few studies that compare the differences in blood loss and haemodynamic changes between sPNL and mPNL; thus, it was decided that the effect and the flushing fluid absorption level associated with the two different surgical methods should be analysed. In addition, there are few studies on the haemodynamic and metabolic changes that occur due to mPNL, and there is a lack of studies that compare the haemodynamic and metabolic changes between ESMS-mPNL and non-ESMS-mPNL; thus, we aimed to explore the effects and the fluid absorption levels associated with the two surgical procedures.

In the present retrospective study, our data indicated the following. (a) Compared with that of sPNL, the operation time of mPNL was significantly longer, and the degree of pain, hospitalization time, and return to work time were significantly reduced. Additionally, in the mPNL group, more tubeless procedures were performed, and the amount of haemoglobin loss was slightly reduced. (b) The results confirmed that there was no difference in fluoroscopy time, complication rate, stone-free rate, or CIRF rate between the mPNL and sPNL groups. (c) The ESMS-mPNL group had a significantly longer irrigation time and a smaller volume of fluid absorbed than the non-ESMS-mPNL group (but these values were clinically comparable), with markedly reduced volume of irrigation fluid, blood loss, haemoglobin loss, degree of pain, hospitalization time, and return to work time. (d) There was no difference in the operation time, complication rate, stone-free rate, CIRF rate or number of tubeless procedures between the non-ESMS-mPNL and ESMS-mPNL subgroups. Our study provides insights that mPNL is more effective and safer than sPNL for managing renal calculi with a diameter of 20–40 mm. However, sPNL is associated with a longer operative time. In addition, ESMS-mPNL is a better method for managing renal calculi than non-ESMS-mPNL.

From a technical perspective, mPNL has more advantages and greater safety for the treatment of kidney stones. First, mPNL uses a smaller percutaneous catheter than sPNL, so the renal parenchyma is less damaged. On the other hand, although smaller renal tubules may hinder the fragmentation and removal of stones, research has shown that mPNL has an obvious advantage in managing renal calculi with diameters of 20–40 mm (21, 22).

Although different definitions of operation time were used in the trials, our summary analysis showed that the operation time of mPNL was obviously longer than that of sPNL. This difference may be due to the narrower field of view of the micro endoscope and the need to break the stones into smaller pieces to remove the pieces through a smaller channel. Moreover, the larger treatment range provides more options for lithotripsy.

The hospitalization and return-to-work times associated with mPNL were shorter than those associated with sPNL. The possible reason is that patients who undergo mPNL have less postoperative discomfort and are more likely to undergo a tubeless surgery (23, 24). The placement of the postoperative nephrostomy tube usually depends on the size of the renal tubules (25); therefore, mPNL is more likely to be completed without a nephrostomy. Previous studies compared the postoperative discomfort between patients treated with mPNL and patients treated with sPNL (17). One tool for analysing surgical discomfort is the VAS for pain analysis (26). In our retrospective study, the VAS was used after 12 h. Although mPNL patients showed significant improvement in their VAS after 12 h, it was uncertain whether this advantage was due to the use of smaller catheters or the omission of nephrostomy tubes.

The volume of irrigation fluid during the operation was significantly lower in the ESMS-mPNL subgroup than in the non-ESMS-mPNL subgroup (*P* < 0.0001). The volume of fluid absorbed during ESMS-mPNL decreased significantly compared to the non-ESMS-mPNL group, and the endoscopic surgical monitoring system might promote better fluid absorption during ESMS-mPNL than during non-ESMS-mPNL. Liquid absorption mainly depends on the flushing pressure and the length of the equipment. Although the non-ESMS-mPNL group absorbed more fluid, it may not be enough to improve the haemodynamic imbalance during surgery.

The advantages of ESMS-mPNL over non-ESMS-mPNL include reduced bleeding, fewer complications, less postoperative pain, a shorter hospitalization time and shorter return to work time, and the main disadvantage is that the operation time and irrigation time are longer. A possible explanation for this is that ESMS provides early real-time monitoring and a timelier warning of irrigation fluid absorption and blood loss to make endoscopic surgical procedures safer for patients.

This study is not without limitations and shortcomings. First, this is a retrospective analysis within a single research institution. Second, mPNL was performed using two different procedures. Third, a two-dimensional calculation of stone size was not obtained. In addition, multicentre, large-scale randomization studies should be performed to further verify the above conclusions, and these studies would increase the statistical significance.

## Conclusion

mPNL and ESMS-mPNL are excellent methods for the treatment of renal stones. ESMS-mPNL is a newer mPNL technique with good efficacy and reduced morbidity and hospitalization times, which benefit patients and improve national health costs. The safety profile of ESMS-mPNL suggests the utilization of ESMS-mPNL for the treatment of renal calculi may be beneficial. Prospective studies are needed to further understand this.

## Data Availability

The datasets presented in this study can be found in online repositories. The names of the repository/repositories and accession number(s) can be found in the article/Suplementary Material.

## Footnote

The charging standard for this device has been established, RMB 130 per hour. The suggested retail price of the machine is RMB 200000. The average cost-benefit ratio was 1.20 ± 0.24. Available at http://www.gsakyx.cn/index.html.
