# Corrigendum: Mini-percutaneous nephrolithotomy with an endoscopic surgical monitoring system for the management of renal stones: A retrospective evaluation

**DOI:** 10.3389/fsurg.2022.999166

**Published:** 2022-08-10

**Authors:** Huiming Gui, Hanzhang Wang, Dharam Kaushik, Ronald Rodriguez, Zhiping Wang

**Affiliations:** ^1^ Department of Urology, Lanzhou University Second Hospital, Lanzhou. China; ^2^ Lanzhou University Second Hospital, Lanzhou, China; ^3^ The University of Texas Health Science Center at San Antonio, San Antonio, TX, United States

**Keywords:** standard percutaneous nephrolithotomy, mini-percutaneous nephrolithotomy, endoscopic surgical monitoring system, renal calculus, retrospective study

A corrigendum on Mini-percutaneous nephrolithotomy with an endoscopic surgical monitoring system for the management of renal stones: A retrospective evaluation by Gui H, Wang H, Kaushik D, Rodriguez R and Wang Z (2022). Front. Surg. 9:773270. doi: 10.3389/fsurg.2022.773270

In the published article, there was an error caused by incorrect writing. A correction has been made to the Abstract of Section. This sentence previously stated:

“… however, the patients in the ESMS-mPNL group had significantly longer operation times than those in the non-ESMS-mPNL subgroup, along with marked reductions in irrigation fluid absorption, blood loss, haemoglobin loss, 12 h postoperative VAS score, mean hospitalization time, and return to work time.”

The corrected sentence appears below:

“… however, the patients in the ESMS-mPNL group had significantly longer irrigation times than those in the non-ESMS-mPNL subgroup, along with marked reductions in irrigation fluid, blood loss, haemoglobin loss, 12 h postoperative VAS score, mean hospitalization time, and return to work time.”

In the published article, there was an error in the legend for [Table 4] as published. [Caused by incorrect writing].

The corrected [Table 4] and its caption **[Comparison of operative data and complications for Non-ESMS-mPNL vs ESMS-mPNL groups.] appear below.

**Table 4:** Comparison of operative data and complications for Non-ESMS-mPNL vs ESMS-mPNL groups.

Data	Non-ESMS-mPNL (*n* = 46)	ESMS-mPNL (*n* = 46)	*P* value
Operation time (min.), mean ± SD	66.1 ± 6.2	68.2 ± 5.6	0.090
Irrigation time (min)	42.2 ± 14.1	52.0 ± 18.3	0.005
Volume of irrigation fluid (ml)	1651.9 ± 631.4	1245.6 ± 548.2	0.001
Volume of fluid absorbed (ml)	712 ± 95	502 ± 102	<0.001
Blood loss (ml)	142.1 ± 93.54	82.2 ± 41.2	<0.001
Hemoglobin loss (mg/dl)	1.21 ± 0.78	1.02 ± 0.63	0.044
VAS score postop 12 h	1.95 ± 0.56	1.66 ± 0.42	0.005
Complications rate			
Clavien 1	2 (4.8)	2 (3.2)	0.996
Clavien 2	–	–	
Clavien 3	–	–	
Clavien 4	–	–	
Mean hospitalization time (hour), mean ± SD	53.82 ± 13.48	47.31 ± 12.04	0.017
Stone-free rate (1. month)	41 (89.1)	42 (90.3)	0.731
CIRF rate (%)	2 (4.3)	1 (2.2)	0.125
Return to work time (day), mean ± SD	12.06 ± 3.21	9.87 ± 2.76	0.001
Tubeless procedure (%)	18 (39.1)	16 (34.8)	0.670

In the published article, there were some errors caused by incorrect writing.

A correction has been made to the Results of Section. This sentence previously stated:

“[A longer irrigation time (52.0 ± 18.3 vs. 42.2 ± 14.1 min) and a larger volume of absorbed fluid (712 ± 95 vs. 502 ± 102 ml) were observed in the patients in the ESMS-mPNL group compared with those in the non-ESMS-mPNL group (*P* = 0.005 and *P* < 0.001, respectively).]”

The corrected sentence appears below:

“[A longer irrigation time (52.0 ± 18.3 vs. 42.2 ± 14.1 min) and a smaller volume of absorbed fluid (502 ± 102 vs. 712 ± 95 ml) were observed in the patients in the ESMS-mPNL group compared with those in the non-ESMS-mPNL group (*P* = 0.005 and *P* < 0.001, respectively).]”

Two corrections have been made to the Discussion of Section. This sentence previously stated:

“[The ESMS-mPNL group had a significantly longer irrigation time and a larger volume of fluid absorbed than the non-ESMS-mPNL group (but these values were clinically comparable),]”

The corrected sentence appears below:

“[The ESMS-mPNL group had a significantly longer irrigation time and a smaller volume of fluid absorbed than the non-ESMS-mPNL group (but these values were clinically comparable),]”

This sentence previously stated:

“[The volume of fluid absorbed during ESMS-mPNL increased significantly compared to the non-ESMS-mPNL group, and the endoscopic surgical monitoring system might promote better fluid absorption during ESMS-mPNL than during non-ESMS-mPNL.]”

The corrected sentence appears below:

“[The volume of fluid absorbed during ESMS-mPNL decreased significantly compared to the non-ESMS-mPNL group, and the endoscopic surgical monitoring system might promote better fluid absorption during ESMS-mPNL than during non-ESMS-mPNL.]”

We apologize for this mistake and declare that this correction will not change the scientific conclusion of this article. The original article has been updated.

